# The Role of Cold Inducible RNA-Binding Protein in Cardiac Physiology and Diseases

**DOI:** 10.3389/fphar.2021.610792

**Published:** 2021-02-24

**Authors:** Peng Zhong, Jianye Peng, Zhouyan Bian, He Huang

**Affiliations:** ^1^ Department of Cardiology, Renmin Hospital of Wuhan University, Wuhan, China; ^2^ Cardiovascular Research Institute of Wuhan University, Wuhan, China; ^3^ Hubei Key Laboratory of Cardiology, Wuhan, China; ^4^ Department of Cardiovascular Medicine, The second Affiliated Hospital of University of South China, Hengyang, China

**Keywords:** CIRP, cardiac electrophysiology, cardiac diseases, cell apoptosis, cold inducible RNA binding protein

## Abstract

Cold-inducible RNA-binding protein (CIRP) is an intracellular stress-response protein that can respond to various stress conditions by changing its expression and regulating mRNA stability. As an RNA-binding protein, CIRP modulates gene expression at the post-transcriptional level, including those genes involved in DNA repair, cellular redox metabolism, circadian rhythms, telomere maintenance, and cell survival. CIRP is expressed in a large variety of tissues, including testis, brain, lung, kidney, liver, stomach, bone marrow, and heart. Recent studies have observed the important role of CIRP in cardiac physiology and diseases. CIRP regulates cardiac electrophysiological properties such as the repolarization of cardiomyocytes, the susceptibility of atrial fibrillation, and the function of the sinoatrial node in response to stress. CIRP has also been suggested to protect cardiomyocytes from apoptosis under various stress conditions, including heart failure, high glucose conditions, as well as during extended heart preservation under hypothermic conditions. This review summarizes the findings of CIRP investigations in cardiac physiology and diseases and the underlying molecular mechanism.

## Introduction

Cold-inducible RNA binding protein (CIRP) was first discovered two decades ago. It came to the attention of researchers because its expression was induced after cells were exposed to a moderate cold shock. Later studies showed that the expression of CIRP could also be regulated by hypoxia, UV radiation, glucose deprivation, heat stress, and H_2_O_2_, suggesting that CIRP is a general stress-response protein (Zhong and Huang, 2017). Depending on the developmental stage, cell type, and stress-conditions, CIRP can accumulate in the nucleus, in the cytoplasm, or both of these cellular compartments (Zhu et al., 2016). A tremendous amount of research on CIRP has revealed its role in the regulation of a variety of cellular stress responses, including cell proliferation, cell survival, the circadian clock, telomerase maintenance, stress adaptation, and tumor formation and progression (Haley et al., 2009; Yang et al., 2010; Morf et al., 2012; Chang et al., 2016; Zhang et al., 2016).

As an RNA-binding protein, CIRP modulates mRNA stability at the post-transcriptional level through directly binding the 3′-UTR of its targets in the cytosol (Yang et al., 2010) (Yang et al., 2006; Zhong and Huang, 2017). Studies have demonstrated that the 3′-UTR binding sites of CIRP are enriched within 100 nucleotides upstream of the polyadenylation sites, and UU and UUU are the possible core recognition sequences of CIRP (Xia et al., 2012; Liu et al., 2013). CIRP consists of an N-terminal RNA recognition motif (RRM) and a C-terminal arginine-rich region. The x-ray crystal structure for the RRM of CIRP was recently reported, and four residues were identified as being likely involved in protein-nucleic acid interactions. These findings may help to serve as a foundation for biophysical studies of this RNA-binding protein and structure-based drug-design efforts for targeting CIRP in pathological conditions, where CIRP level is elevated and contributes to disease progression (Coburn et al., 2017).

In addition to its function in intracellular space, CIRP is found in extracellular space in various inflammatory conditions. Extracellular CIRP could act as a pro-inflammatory factor to trigger inflammation (Qiang et al., 2013; Zhong et al., 2020). Extracellular CIRP has been demonstrated to bind direly to TLR4/MD2 and TREM-1 and activate these inflammatory receptors to trigger inflammation (Qiang et al., 2013; Denning et al., 2020). The critical pro-inflammatory role of extracellular CIRP suggests that targeting CIRP may have therapeutic potential in controlling inflammatory diseases.

The heartbeat provides a force that pumps oxygenated and deoxygenated blood to and from peripheral tissues. This process depends on the orderly activation and recovery of electrical excitation through the myocardium. Understanding the mechanism underlying their generation and maintenance requires knowledge of the ionic contributions to the cellular action potential. Cardiac action results from the sequential opening and closing of the ion channel proteins that span the plasma membrane of individual myocytes. Its conduction through the heart depends on the electrical coupling between these cells, mediated by gap junctions. Disruptions of these cellular ion channels can lead to arrhythmias, which constitute a major public health problem. Recent studies further implicate the important role of CIRP in cardiac electrophysiology by post-transcriptional regulating the expression of specific ion channels and molecular targets. CIRP is also a protective factor against cell apoptosis in cardiomyocytes in response to various stress conditions. This article reviews findings on CIRP in cardiac physiology and diseases and the underlying molecular mechanism.

## The Role of CIRP in Cardiac Electrophysiology

### CIRP Regulates the Function of the Sinoatrial Node (SAN) in Response to Stress

A very recent study has further demonstrated the critical role of CIRP in regulating the function of SAN (Xie et al., 2020b). Used telemetric ECG monitoring, the study found that CIRP KO rats showed an excessive acceleration of heart rate under isoprenaline stimulation, compared to wild-type (WT) rats. Isolated SAN cells also consistently showed a faster spontaneous firing rate under isoprenaline stimulation, as demonstrated by Patch-clamp analysis and confocal microscopic Ca^2+^ imaging technic.

Mechanistic studies found that cAMP concentration, which is a key mediator of pacemaker activity, was higher in CIRP-KO SAN tissues than in WT SAN tissues. Further RNA sequencing and qRT-PCR analysis of single cells revealed that PDE4 (phosphodiesterase 4), which controls cAMP degradation, was significantly decreased in CIRP-KO SAN cells, suggesting that reduced PDE4 expression may contribute to the increased cAMP concentration. Interestingly, the pharmacological inhibition of PDE4 abolished the difference in beating rate caused by CIRP deficiency, suggesting the role played by PDE4 in mediating CIRP deficiency-related firing rate in SAN cells upon isoprenaline stimulation. Moreover, the authors further demonstrated that CIRP could bind to and stabilize the mRNA of PDE4, thereby regulating the protein expression of PDE4 at post-transcriptional level.

These results demonstrate the physiological role of CIRP in regulating the firing rate of SAN in response to stress by acting as an mRNA stabilizer of PDE4 and controlling the cAMP concentration in SAN cells. The physiological impact may be that CIRP may prevent the heart from overreacting to stressors, and CIRP may be a promising new potential target for heart rate control.

### CIRP Regulates Atrial Repolarization and the Susceptibility to Atrial Fibrillation Onset

CIRP was also recently reported to play an important role in regulating atrial electrophysiology, modulating the susceptibility of atrial fibrillation (AF), the most common sustained arrhythmia (Xie et al., 2020a). In this study, CIRP KO rats had a shortened atrial effective refractory period (AERP). They showed increased susceptibility to AF induction, following programmed atrial stimulation both *in vivo* and in isolated Langendorf-perfused heart compared to wild-type rats. Consistent with *in vivo* study, atrial cardiomyocytes from CIRP KO rats also showed shortened action potential duration (APD), coupled with enhanced I_Kur_ and I_to_ current. As Kv1.5 mediates the ultra-rapid delayed rectifier potassium current (I_Kur_) in atrial myocytes and is critical for atrial repolarization and Kv4.2/4.3 channels conduct the fast transient outward current (Ito) of the cardiac action potential (AP) in the myocardium.

Interestingly, all the protein levels of these channels were upregulated in the atrium of CIRP KO rats, compared to wild-type rats. Therefore, the increased protein level of Kv4.2/4.3 and Kv1.5 in atria tissue contributed to the enhanced I_Kur_ and I_to_ current in atrial cardiomyocytes from CIRP-deficient rats. Further mechanistic studies have demonstrated that CIRP could posttranscriptionally and negatively regulate atrial Kv1.5 and Kv4.2/4.3 channels by binding to and targeting their 3′UTRs of their mRNAs. Atria-specific CIRP delivery through an AAV9-mediated gene therapy could prolong the AERP and prevent AF occurrence in CIRP KO rats, coupled with a decreased protein level of Kv1.5 and Kv4.2/4.3 in the atrium (Xie et al., 2020a). This study demonstrates the important role of CIRP in atrial electrophysiology and in initiating AF onset by directly regulating the expression of the Kv1.5 and Kv4.2/4.3 channels posttranscriptionally, indicating that CIRP could be a promising potential target for interventions in AF.

### CIRP Regulates Ventricular Repolarization

Another study showed that CIRP could also regulate ventricular myocardial repolarization by targeting transient outward potassium channels (Li et al., 2015). In this study, the authors found that rats with globe CIRP knockout had structurally and functionally normal hearts. In resting conditions, CIRP null mice showed normal RP interval, RR interval, and QRS wave, as compared to wild-type mice. However, the rate-corrected QT (QTc) interval was shorter in resting CIRP KO mice. In addition, ventricular cardiomyocytes from CIRP KO mice showed an abbreviated action potential duration, coupled with increased transient outward potassium current (Ito). The mechanistic study revealed that CIRP protein can selectively bind to KCND2 and KCND3 mRNA, which encode the functional α-subunits of the voltage-gated potassium channels Kv4.2 and Kv4.3, conducting the fast transient outward current (Ito) of the cardiac AP in the myocardium. CIRP deficiency can upregulate the protein levels of Kv4.2 and Kv4.3 without changing the transcriptional activity of their respective genes, suggesting that CIRP deficiency facilitates translation in a post-transcriptional manner.

These data demonstrate the physiological role of CIRP in ventricular repolarization. Considering that abnormal ventricular repolarization is linked to various arrhythmic manifestations, the constitutive expression of CIRP in the heart and the CIRP-dependency of cardiac repolarization, may imply the critical involvement of CIRP in ventricular electrophysiology and arrhythmogenesis.

**FIGURE 1:**
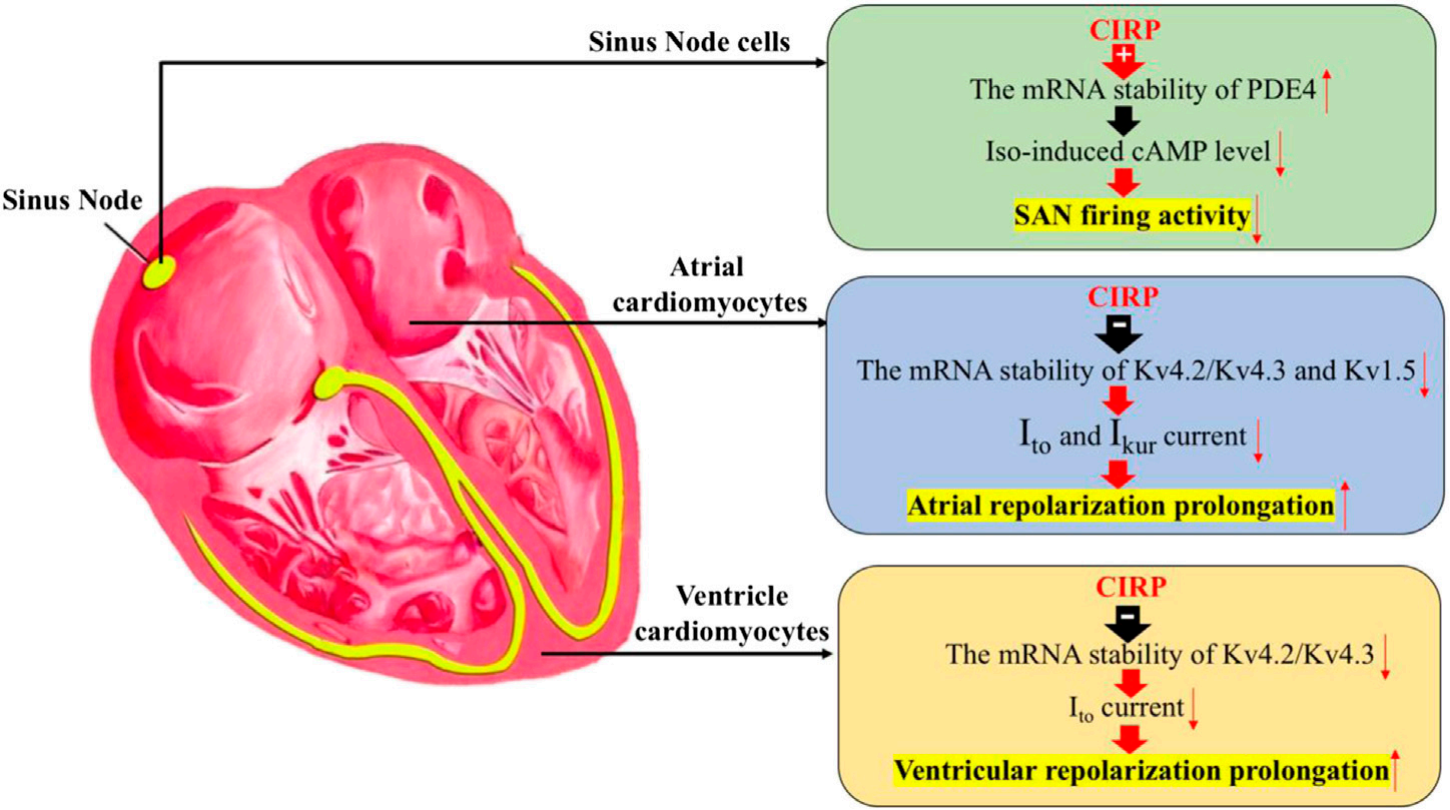
The effects of CIRP in cardiac electrophysiology. Firstly, CIRP can suppress the firing activity of the sinus atrial node (SAN) cells by positively regulating the mRNA stability of PDE4, which is the key enzyme for the degradation of the isoprenaline-induced cAMP level. Secondly, CIRP can delay the atrial repolarization by negatively influencing the mRNA stability of Kv4.2/Kv4.3 and Kv1.5, which codes for the repolarization current of I_to_ and I_Kur_. Thirdly, CIRP can delay the ventricle repolarization by negatively influencing the mRNA stability of Kv4.2/Kv4.3, which codes for the repolarization current of I_to_. PDE4: phosphodiesterase four; Iso: Isoprenaline.

These studies indicate the important role of CIRP in regulating the electrophysiological properties of the heart, including cardiac pacemaker activity in response to stress, the repolarization phase of atrial and ventricular cardiomyocytes by stabilizing the mRNA of PDE4 in the sinus node cells and destabilizing the mRNA of Kv4.2/Kv4.2 in both atrial and ventricular cardiomyocytes, as well as the mRNA of Kv1.5 specifically in atrial cells (Figure 1).

## The Role of CIRP in Various Cardiac Stress Conditions

### CIRP Protects Cardiomyocyte From Apoptosis in Heart Failure

Recently, our group found that CIRP is downregulated in heart failure. The downregulation of CIRP then rendered cardiac cells prone to apoptosis in heart failure (HF) (Chen et al., 2019). In this study, we found that CIRP protein levels were significantly reduced in heart samples from both patients with HF and mice with post-myocardial infarction (post-MI), compared to that in the control samples, suggesting a possible involvement of CIRP in the HF development. In addition, *in vitro* experiments have shown that CIRP silencing promoted more cell apoptosis in cardiac cells under H_2_O_2_ stimulation. These results imply the role of CIRP in HF development, possibly by regulating cell surviving capability in response to stress injury.

### CIRP Protected Cardiac Apoptosis and Dysfunction During Extended Heart Preservation Under the Hypothermic Condition

Long-term hypothermic heart preservation during heart transplantation leads to decreased cardiac function. Recently, CIRP was demonstrated to protect cardiomyocytes from apoptosis during extended hypothermic heart preservation (Pan et al., 2020). In this study, CIRP gene-modified rats were subjected to various heart preservation times, followed by evaluating cardiac function using the Langendorff apparatus and histological and molecular study. With 6-h preservation, no significant difference was found in cardiac functions and histological changes among different rat species, including CIRP KO rats, CIRP TG (Transgene) rats, and wild-type rats. However, after 12 h of preservation, the hearts of CIRP KO rats showed more cell apoptosis and worse cardiac function, while CIRP TG rat hearts showed less apoptosis and a better cardiac function. Further mechanistic study revealed that CIRP can posttranscriptionally regulate the protein expression of ubiquinone biosynthesis protein COQ9, an essential component in regulating the ubiquinone (CoQ_10_) biosynthesis. As CoQ10 can promote ATP production and protect cells against oxidative stress, the study detected higher CoQ_10_ concentration, higher ATP concentration, and decreased levels of oxidative markers in the heart tissues of CIRP TG rats, compared to those in WT rats. By contrast, the myocardial concentration of CoQ10 and ATP was significantly decreased, coupled with a decreased protein level of COQ9 in CIRP KO rats than the WT rats. These results suggest that CIRP-mediated CoQ_10_ biosynthesis plays an important role in hypothermic cardioprotection during extended heart preservation. Furthermore, the author showed that administration of a CIRP agonist, called zr17-2 (Coderch et al., 2017), could extend heart preservation with enhanced expression of CIRP, increased CoQ_10_ concentration, and ATP level, as well as promote scavenging of reactive oxygen species. These data demonstrate the protective role of CIRP in extended heart preservation. The pharmacological activation of CIRP by zr17-2 may be a promising strategy in ameliorating heart damage and extending heart preservation during cardiac transport.

### Epigenetic Modification of the CIRP Gene in Cardiomyocytes Under Chronic Hypoxia Contributed to Reduced Cardiac Protection During the Hypothermic Condition

A recent study showed that chronic hypoxia could induce epigenetic modification of CIRP by hypermethylation of CIRP promoter region in cardiomyocytes, resulting in the depression of CIRP expression and the failure of cardiomyocytes in response to cold stress (Liu et al., 2019). The authors also demonstrated that CIRP plays an important role in regulating the ubiquinone biosynthesis pathway, as CIRP can bind to and posttranscriptionally enhance the mRNA expression of COQ6 and COQ9, which play an important role in CoQ_10_ biosynthesis. Hypothermia during cardiopulmonary bypass (CPB) surgery could significantly increase cardiac CIRP expression, which bound to the COQ6 and COQ9 mRNAs and posttranscriptionally enhanced their expression, resulting in increased CoQ10 biosynthesis and cardiac protection. However, the failure of CIRP in response to cold stress in chronically hypoxic myocardium could lead to decreased concentration of CoQ_10_ and impaired cardioprotective effects of hypothermia during CBP. CIRP KO rats also showed downregulation of essential components of the ubiquinone biosynthesis pathway (such as COQ6 and COQ9), decreased concentration of CoQ_10_, decreased ATP production, and aggravated oxidative stress, coupled with the increased myocardial injury during CPB surgery. Overexpression of CIRP or addition of COQ_10_ to the cardioplegic solution could significantly improve the cardioprotective effect for chronically hypoxic myocardium during CPB surgery, suggesting that the hypoxia-CIRP-COQ_10_ axis has a key role in this process.

These results uncover a new mechanism for the downregulation of CIRP in cardiomyocytes under chronic hypoxia conditions. They demonstrated that CIRP is a key regulator of the CoQ_10_ biosynthesis pathway in cardiomyocytes. These new mechanisms have clinical significance, and may inform the clinical phenomenon that chronic hypoxia-induced suppression of CIRP is likely responsible for the high risk of several myocardial injuries after cardiac surgery. Strategies targeting this pathway may have therapeutic potential for reversing decreased hypothermia-induced heart protection during CBP in patients with complicated chronic hypoxia conditions.

### CIRP Protected Cell Apoptosis in Cardiac Cells Under High Glucose Conditions

A recent study revealed the protective role of CIRP in cardiac injury in response to high glucose concentration *in vitro* (Zhao et al., 2018). This study used high glucose-stimulated H9C2 cardiac cells as an *in vitro* cell model. They showed that under high glucose conditions, the silencing of CIRP resulted in more cell death and apoptosis, coupled with increased ROS generation and elevated inflammatory cytokines expression. This suggests the protective role of CIRP in cardiac cells under high-glucose conditions and implicates a possible role of CIRP in diabetic cardiomyopathy. Although this study is just an *in vitro* study and no animal experiment was performed to further evaluate the involvement of CIRP in the diabetic heart, this pilot gives us a possible clue to further investigate the role of CIRP in mediating diabetic cardiomyopathy.

**FIGURE 2:**
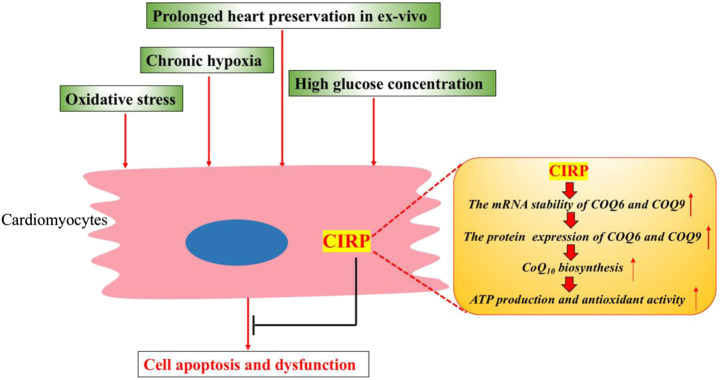
CIRP protects cardiomyocytes from apoptosis and dysfunction under various stress conditions. CIRP can upregulate the CoQ10 biosynthesis by positively and posttranscriptionally regulating the protein expression of ubiquinone biosynthesis protein COQ9 and COQ6. CoQ10 could promote mitochondrial metabolism, leading to higher ATP production and improved antioxidant activity.

These studies consistently demonstrate the essential role of CIRP in protecting cardiomyocytes from apoptosis under various stress conditions, including diabetes, chronic hypoxia, oxidative stress, and prolonged heart preservation. The protective mechanism for CIRP could relate to its role in upregulating the CoQ10 biosynthesis pathway by posttranscriptionally stabilizing the mRNA of ubiquinone biosynthesis protein COQ9 and COQ6 in cardiomyocytes (Figure 2).

## Future Perspective

Pharmacological intervention remains the mainstay of arrhythmia management, despite significant interventional progress, including ablation and device therapy. Current known anti-arrhythmic drugs elicit their function via their actions on different components of the cardiac action potential, either work as Na^+^ channel blocker (class I drugs), β-adrenergic inhibition (class II drugs), K^+^ channel block (class III drugs), or L-type Ca^2+^ channel inhibition (class IV drugs) (Barton et al., 2020). However, these pharmacological agents possess significant cardiac and extracardiac side effects, the most concerning of which are proarrhythmic properties. New treatment strategies are, therefore, needed to overcome these challenges. The electrophysiological properties of CIRP, regulating ion channel expression, and subsequently modulating cardiac action potential duration, may provide a new treatment strategy for arrhythmia management. The remarkably reduced atrial fibrillation induction by AAV9-mediated CIRP overexpression in CIRP KO mice demonstrated a therapeutic potential of targeting CIRP in atrial fibrillation. Further studies need to evaluate the anti-arrhythmia effects of targeting CIRP in various experimental arrhythmia animal models.

The *in vitro* protective effects of CIRP on cardiac apoptosis under high glucose concentration and oxidative stress conditions should be further evaluated in animal models, such as animals suffering from diabetes and heart failure. The CIRP agonist---zr17-2, could be utilized to test the effects of boosting CIRP expression in various disease conditions and will facilitate the translation of CIRP-based therapeutic strategies.

The interesting finding, that CIRP released in extracellular space can act as a damage-associated molecular pattern (DAMP) to trigger an inflammatory response in multiple cells, including macrophages and vascular endothelial cells by activating TLR4/MD2 signaling pathways, as well as TREM-1 signaling pathways, may have important implications in cardiac diseases. It has been shown that serum CIRP level was increased in patients and experimental models with sepsis or hemorrhage and contributed to lethal cytokine storm in sepsis (Qiang et al., 2013; Zhong et al., 2020). The serum level of CIRP was also found to be increased in several acute organ ischemia/reperfusion (I/R) mouse models involved in liver, kidney, brain, as well as intestine, and organ injury could be significantly attenuated by CIRP knockout or blockage (Zhou et al., 2014; Godwin et al., 2015; Cen et al., 2016; Yang et al., 2016; Cen et al., 2017). These studies suggest that under situations of inflammation and tissue injury, CIRP may be released actively or passively from the intracellular space into the extracellular space, and extracellular CIRP acts as a pro-inflammatory factor to further trigger and amplify inflammation in immune cells or non-immune cells. Therefore, the role of CIRP may also be implicated in acute myocardial infarction (MI) or myocardial I/R injury, or other cardiac diseases associated with chronic inflammation, which need further investigation.
